# Supplementary material for: A Critical View of the Application of the APEX Software (Aqueous Photochemistry of Environmentally-Occurring Xenobiotics) to Predict Photoreaction Kinetics in Surface Freshwaters
Source: Molecules. 2019 Dec 18;25(1):9. doi: 10.3390/molecules25010009 (PMC7017383; doi:10.3390/molecules25010009)
Supplement: Supplementary file 1 [file molecules-25-00009-s001.zip › 3.2.4_gcc-4.4.0.zip!/3.2.4_gcc-4.4.0/3.2.4_gcc-4.4.0/doc/octave/html/liboctave/Differential-Algebraic-Equations.html]

Differential Algebraic Equations - Octave C++ Classes


Next: Error Handling,
Previous: Ordinary Differential Equations,
Up: Top


---

## 12 Differential Algebraic Equations

— : **DAE** (void)  
— : **DAE** (int n)  
— : **DAE** (const ColumnVector &x, double time, DAEFunc &f)  
— : **DAE** (const ColumnVector &x, ColumnVector &xdot, double time, DAEFunc &f)

— : ColumnVector **deriv** (void)

— : virtual void **initialize** (const ColumnVector &x, double t)  
— : virtual void **initialize** (const ColumnVector &x, ColumnVector &xdot, double t)

— : ColumnVector **integrate** (double t)

— : Matrix **integrate** (const ColumnVector &tout, Matrix &xdot\_out)  
— : Matrix **integrate** (const ColumnVector &tout, Matrix &xdot\_out, const ColumnVector &tcrit)
